# Antisynthetase Syndrome With Classic Features and Anti‑PL‑7 Positivity: A Rare Immunologic Variant

**DOI:** 10.7759/cureus.93503

**Published:** 2025-09-29

**Authors:** Karen S Arrazola-Mendoza, Hugo E González-Chávez, Francisco De la Peña-Camacho, Emmanuel Reyes-Ferreira, Ulises Gomez-Alvarez

**Affiliations:** 1 Internal Medicine, General Hospital of the Institute of Security and Social Services of State Workers of Querétaro, Queretaro, MEX

**Keywords:** anti–pl-7, antisynthetase syndrome, idiopathic inflammatory myopathies, interstitial lung disease, myositis

## Abstract

Antisynthetase syndrome (ASyS) is a rare and heterogeneous subtype of idiopathic inflammatory myopathies, characterized by the presence of autoantibodies against aminoacyl-tRNA synthetases. Among these, anti-PL-7 antibodies are infrequent and associated with variable clinical expression. We present the case of a 71-year-old woman who developed progressive proximal muscle weakness. Physical examination revealed a heliotrope rash and mechanic’s hands. Laboratory tests showed markedly elevated creatine kinase levels, and chest computed tomography findings were consistent with interstitial lung disease. Serological testing confirmed the presence of anti-PL-7 antibodies, leading to the diagnosis of ASyS. This case highlights the diagnostic challenges of ASyS, particularly when rare autoantibodies such as anti-PL-7 are involved. Recognition of characteristic clinical features and serological findings is essential for timely diagnosis and appropriate management.

## Introduction

Idiopathic inflammatory myopathies (IIMs) are a heterogeneous group of systemic autoimmune disorders characterized by both muscular and extramuscular manifestations [1]. The incidence of IIMs ranges from 0.2 to 2 cases per 100,000 individuals per year, with a prevalence of 2 to 25 per 100,000 [2]. Five major subtypes of IIMs are currently recognized: dermatomyositis (DM), polymyositis, inclusion body myositis, immune-mediated necrotizing myopathy, and antisynthetase syndrome (ASyS), which accounts for approximately 11% [3].

ASyS is a multisystem disease that primarily affects the muscles, joints, skin, and lungs [4]. Its hallmark feature is the presence of autoantibodies directed against aminoacyl-tRNA synthetases. Among these, anti-Jo-1 antibodies are the most common, detected in 20-30% of cases. Non-Jo-1 ASyS is associated with rarer antibodies, including anti-PL-7, anti-PL-12, anti-EJ, anti-OJ, anti-KS, anti-Ha, and anti-Zo, each with a prevalence of less than 5%, collectively accounting for up to 40% of ASyS cases [5]. Each autoantibody is linked to distinct clinical phenotypes and disease severity. The diagnosis and management of ASyS remain challenging due to the multisystemic nature of its clinical presentation [6]. Limited data are available on the clinical features of myositis associated with anti-threonyl-tRNA synthetase (anti-PL-7) antibodies, likely due to its rarity (present in only about 5% of all IIM cases) [7].

## Case presentation

**Figure 1:**
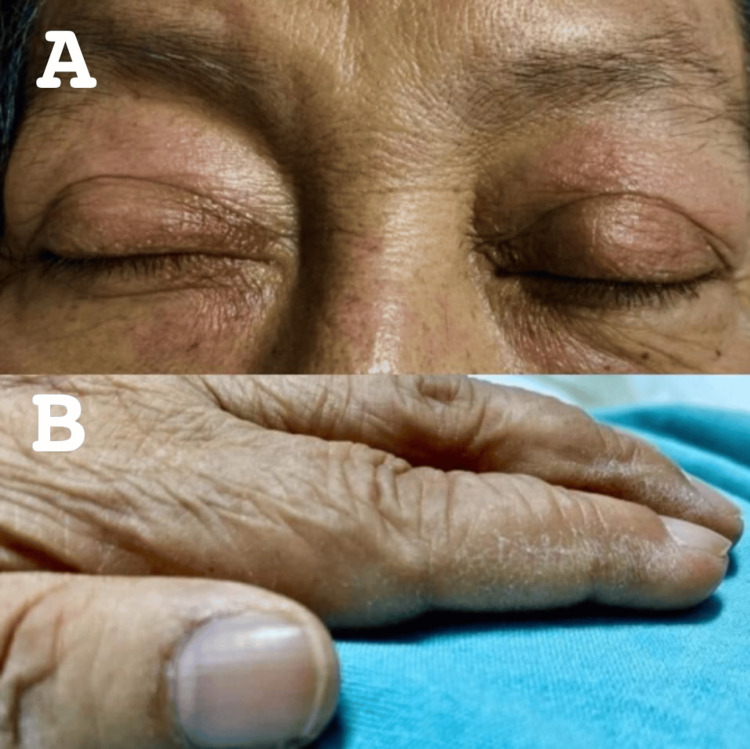
Dermatologic findings on physical examination A) Heliotrope rash characterized by a violaceous discoloration of the eyelids. B) Mechanic’s hands presenting as hyperkeratotic, fissured, and erythematous changes over the lateral aspects of the fingers and palms.

A 71-year-old woman with a medical history of long-standing diabetes mellitus and hypertension was admitted to the hospital with a two-month history of progressive muscle symptoms. Initially, she developed myalgias and symmetric proximal weakness in the lower limbs, resulting in loss of ambulation. Subsequently, similar weakness appeared in the upper limbs, leading to hospital admission. Physical examination revealed heliotrope rash and mechanic’s hands (Figure 1).

Muscle strength, assessed by the Daniels scale, was 1/5 in the lower limbs and 2/5 in the upper limbs. No other significant findings were noted. Laboratory tests revealed elevated creatine kinase (CK) levels (Table 1).

**Table 1:** Laboratory results and normal ranges The results from laboratories with normal ranges are shown, revealing a significant elevation of CK.

Laboratories	Results	Normal ranges
Hemoglobin	14.5 g/dL	13.0 - 16.0 g/dL
Hematocrit	41.6%	39.0 - 48.0%
Platelets	380 x 10^3 ^/uL	150 - 450 x 10^3^ /uL
Leukocytes	10.49 x 10^3^ /uL	4.5 - 11.0 x103 /uL
Calcium	9.2 mg/dL	8.4 - 10.2 mg/dL
Phosphorus	5.2 mg/dL	2.5 - 4.5 mg/dL
Chloride	106 mmol/L	98 - 107 mmol/L
Sodium	141 mmol/L	135 - 145 mmol/L
Magnesium	2 mg/dL	1.6 - 2.3 mg/dL
Potassium	4.0 mmol/L	3.5 - 5.1 mmol/L
Lactate dehydrogenase (LDH)	400 u/L	120 - 246 u/L
Creatine kinase	8,000 u/L	30 - 135 u/L

**Figure 2:**
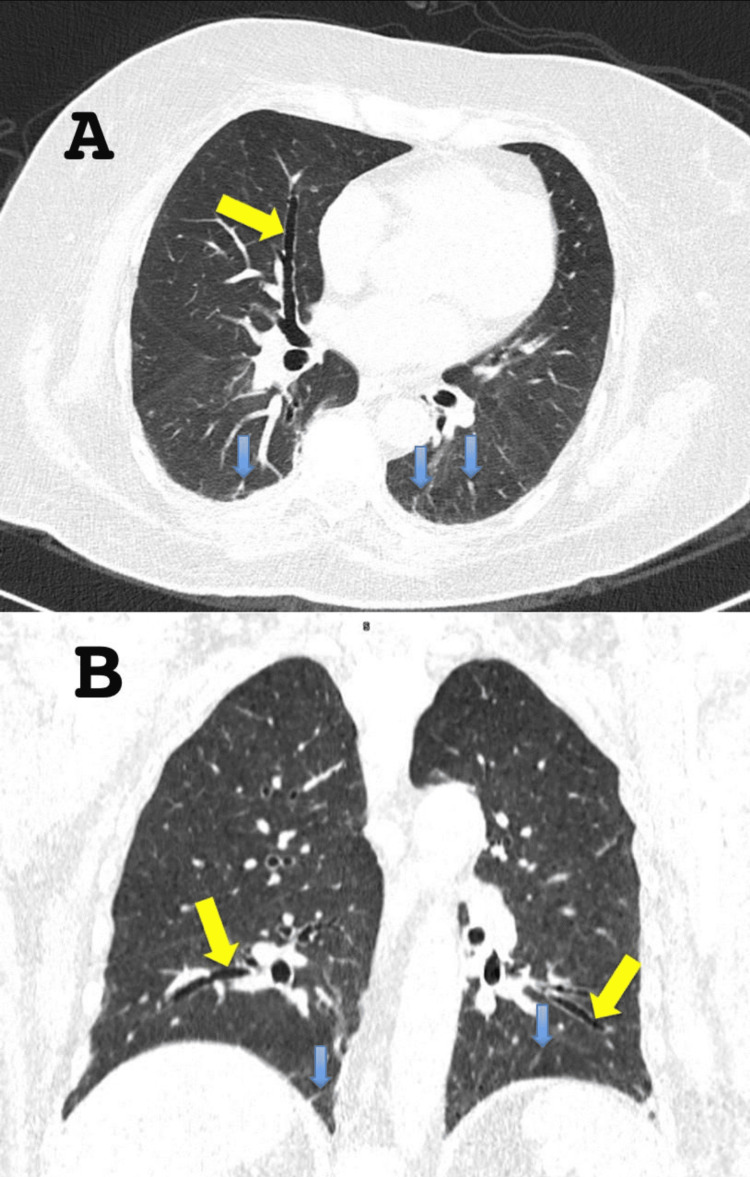
Computed tomography (CT) of the chest in lung window settings (A) Axial view demonstrates interstitial thickening (blue arrow) and cylindrical bronchiectasis (yellow arrow). (B) Coronal view confirms the presence of bilateral interstitial thickening (blue arrow) and cylindrical bronchiectasis (yellow arrow), predominantly in the lower lung zones. These findings are consistent with ILD. ILD: Interstitial lung disease

During hospitalization, her weakness progressed, involving respiratory muscles and leading to dyspnea. Chest computed tomography revealed bilateral apical-predominant interstitial thickening, cylindrical basal bronchiectasis, linear hyperdensities, and increased intrapleural fluid, consistent with interstitial lung disease (ILD) (Figure 2).

**Figure 3:**
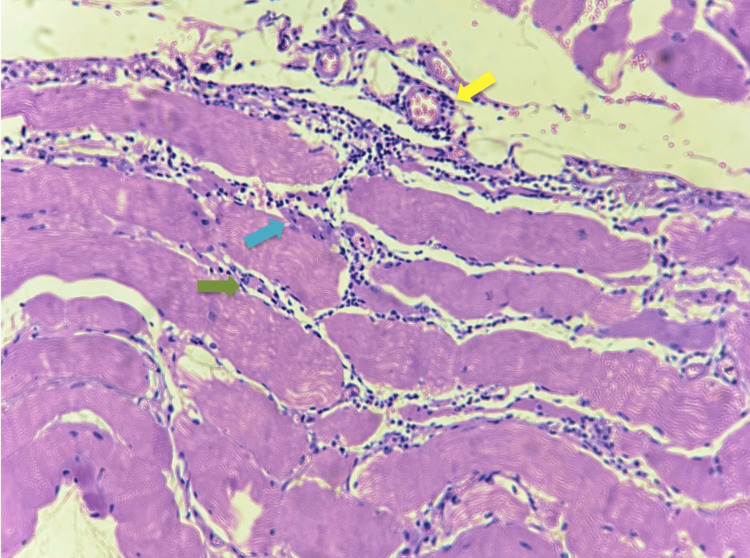
Histopathological findings on muscle biopsy A hematoxylin and eosin-stained section of skeletal muscle demonstrates inflammatory features associated with ASyS. Yellow arrow: focal perivascular inflammatory infiltrate. Blue arrow: perifascicular muscle necrosis. Green arrow: endomysial mononuclear inflammatory infiltrate.

Muscle biopsy showed mononuclear inflammatory infiltrates predominantly in the endomysium with focal perivascular involvement, along with perifascicular necrosis and atrophy. No inclusion bodies were observed (Figure 3). 

An extended myositis antibody panel revealed a positive antinuclear antibody (ANA) with a cytoplasmic pattern at 1:160 and the presence of anti-PL-7 antibodies (Table 2). 

**Table 2:** Myositis autoantibody panel results The patient’s serologic testing revealed positive ANA with a cytoplasmic pattern at a titer of 1:160, as well as positivity for anti–PL-7 autoantibodies. Other antibodies typically associated with IIMs were negative.

Antibody	Result
Anti-Mi2a	Negative
Anti-Mi2b	Negative
Anti-TIF1gamma	Negative
Anti-MDA5	Negative
Anti-NXP2	Negative
Anti-SAE1	Negative
Anti-Ku	Negative
Anti-PM-Scl100	Negative
Anti-SCL75	Negative
Anti-Jo1	Negative
Anti-SRP	Negative
Anti-PL7	Positive
Anti-PL12	Negative
Anti-EJ	Negative
Anti-Oj	Negative
Anti-Ro52	Negative
ANA	1:160 Cytoplasmic pattern

A diagnosis of ASyS was established. The patient received intravenous methylprednisolone pulses (500 mg every 24 hours, three doses) with partial improvement (recovery of muscle strength assessed by the Daniels scale was 3/5 in the lower limbs and 4/5 in the upper limbs) and a decrease in CK levels (Table 3). She was discharged with prednisone 50 mg every 24 hours, methotrexate 15 mg subcutaneous once a week and follow-up in an outpatient clinic to start rituximab.

**Table 3:** CK levels The table displays the progressive decrease in CK levels. CK: Creatine kinase

Date	CK Levels
25/01/2024	8,000 u/L
31/01/2024	3,200 u/L
02/02/2024	1,282 u/L
20/02/2024	841 u/L
26/02/2024	422 u/L

## Discussion

As with most autoimmune diseases, ASyS results from a breakdown in immune tolerance, leading to autoreactive immune responses. It has been hypothesized that tissue injury triggers the release of aminoacyl-tRNA synthetases from damaged cells, initiating an innate and adaptive immune cascade that culminates in immune cell-mediated end-organ damage.

Patients with ASyS often present with features overlapping those of other IIMs. Characteristic skin findings include mechanic’s hands, along with Raynaud’s phenomenon and inflammatory rashes such as heliotrope rash, Gottron’s signs, the V-sign, shawl sign, and malar rash. Articular involvement typically presents as inflammatory arthralgia and non-erosive arthritis. Muscle involvement manifests as significant proximal muscle weakness, often more pronounced in the lower limbs. Serum CK levels are frequently elevated, often exceeding 4,000 U/L [8].

ILD is commonly present at the time of diagnosis and contributes substantially to morbidity and mortality. The most frequently observed radiologic patterns include nonspecific interstitial pneumonia and organizing pneumonia, although usual interstitial pneumonia may also occur [9,10]. In ASyS associated with anti-PL-7 antibodies, the most frequent manifestations are ILD (77%), myositis (75%), and arthritis (56%) [11]. These antibodies are associated with more severe pulmonary involvement. However, the prognostic implications of different antisynthetase autoantibodies remain unclear [12].

Histopathologically, muscle biopsies may demonstrate perifascicular atrophy similar to DM [13]. However, studies from multiple groups have identified perifascicular necrosis, rather than atrophy alone, as the most distinctive histological feature differentiating ASyS from DM [14].

Although ASyS was first described by Marguerie et al. in 1990, the first expert-opinion-based diagnostic criteria were proposed by Connors et al. in 2010, followed by alternative definitions from Solomon et al. in 2011 and Lega et al. in 2015 [15]. Despite these efforts, none of the proposed criteria has been widely validated or adopted. The ongoing EULAR/ACR Classification Criteria for Anti-Synthetase Syndrome (CLASS) project aims to establish data-driven definitions for clinical and serological features [9]. Our patient met several proposed criteria, including clinical and histological evidence of myositis, ILD, mechanic’s hands, heliotrope rash, and anti-PL-7 positivity.

First-line treatment consists of oral or intravenous glucocorticoids. In severe cases, especially with ILD, additional immunosuppressive or biologic therapy is often required [16].

This case highlights the diagnostic complexity of ASyS with anti-PL-7 positivity, a rare and clinically heterogeneous presentation. The diagnosis was confirmed through a combination of clinical observations and laboratory tests. Early serological testing and muscle biopsy were critical in establishing the diagnosis. The rapid progression to respiratory failure highlights the potential severity of this serologic variant.

## Conclusions

ASyS is an uncommon but potentially severe autoimmune disorder that affects multiple organ systems. The diagnosis of this condition is often challenging due to the nonspecific nature of the early clinical manifestations. This case underscores the importance of recognizing a combination of suggestive symptoms, including characteristic dermatologic and muscular findings, in conjunction with specific myositis-associated autoantibodies such as anti-PL-7. Prompt diagnosis and early initiation of immunosuppressive therapy are essential to improve clinical outcomes. Therefore, clinicians should maintain a high index of suspicion in patients presenting with persistent muscular and respiratory symptoms, even in the presence of rare autoantibodies.
